# What defines a synthetic riboswitch? – Conformational dynamics of ciprofloxacin aptamers with similar binding affinities but varying regulatory potentials

**DOI:** 10.1093/nar/gkab166

**Published:** 2021-03-27

**Authors:** Christoph Kaiser, Jeannine Schneider, Florian Groher, Beatrix Suess, Josef Wachtveitl

**Affiliations:** Institute for Physical and Theoretical Chemistry, Goethe-Universität Frankfurt, Max-von-Laue-Straße 8, D-60438 Frankfurt am Main, Germany; Department of Biology, Technische Universität Darmstadt, Schnittspahnstraße 10, D-64287 Darmstadt, Germany; Department of Biology, Technische Universität Darmstadt, Schnittspahnstraße 10, D-64287 Darmstadt, Germany; Department of Biology, Technische Universität Darmstadt, Schnittspahnstraße 10, D-64287 Darmstadt, Germany; Centre for Synthetic Biology, Technische Universität Darmstadt, Darmstadt, Germany; Institute for Physical and Theoretical Chemistry, Goethe-Universität Frankfurt, Max-von-Laue-Straße 8, D-60438 Frankfurt am Main, Germany

## Abstract

Among the many *in vitro*-selected aptamers derived from SELEX protocols, only a small fraction has the potential to be applied for synthetic riboswitch engineering. Here, we present a comparative study of the binding properties of three different aptamers that bind to ciprofloxacin with similar *K*_D_ values, yet only two of them can be applied as riboswitches. We used the inherent ligand fluorescence that is quenched upon binding as the reporter signal in fluorescence titration and in time-resolved stopped-flow experiments. Thus, we were able to demonstrate differences in the binding kinetics of regulating and non-regulating aptamers. All aptamers studied underwent a two-step binding mechanism that suggests an initial association step followed by a reorganization of the aptamer to accommodate the ligand. We show that increasing regulatory potential is correlated with a decreasing back-reaction rate of the second binding step, thus resulting in a virtually irreversible last binding step of regulating aptamers. We suggest that a highly favoured structural adaption of the RNA to the ligand during the final binding step is essential for turning an aptamer into a riboswitch. In addition, our results provide an explanation for the fact that so few aptamers with regulating capacity have been found to date. Based on our data, we propose an adjustment of the selection protocol for efficient riboswitch detection.

## INTRODUCTION

Synthetic biology provides innovative solutions for challenges in a wide range of fields including synthetic genetic circuit design, bio-based materials, bioremediation or diagnostics as well as various therapeutic applications in medicine. Engineered riboswitches are promising tools to explore many of the key questions in these disciplines. They are small and defined *cis-*regulatory elements, can operate protein-independently and allow for fast regulatory responses at different levels of gene regulation (e.g. transcription, translation, splicing (1)). Riboswitches usually consist of an aptamer domain and an expression platform. The aptamer domain couples the specific sensing of a small molecule to the expression of a downstream gene, thus enabling spatial, temporal and dosage-dependent control thereof. The expression platform implements the regulatory mechanism, in most cases transcription termination or sequestration of the ribosomal binding site. However, aptamers can also act without an expression platform as riboswitch by a road blocking mechanism. Such aptamers can be identified *de novo* by an *in vitro* evolution process called SELEX (Systematic Evolution of Ligands by EXponential enrichment), essentially binding any target of choice with high affinity and specificity (2,3). Depending on the nature of the target molecule and the selection protocol, it is possible to obtain aptamers with very different structures, yet similar *K*_D_ values. However, only a few of these have the potential to be further engineered into riboswitches. Interestingly, those sequences are mostly underrepresented in an enriched SELEX pool. Evidently, an *in vivo* screening subsequent to the SELEX process is required for effective identification of candidates with regulating capacity. The short list of examples for successfully engineered riboswitches includes aptamers that bind theophylline (4), neomycin (5) and tetracycline (6) as well as the recent additions of ciprofloxacin- (7) and paromomycin-binding aptamers (8). Several types of engineered riboswitches have already been designed with the theophylline aptamer, such as self-cleaving ribozymes or riboswitches that regulate translation initiation by sequestration of the ribosomal binding site (9). Moreover, it has been applied as transcriptional riboswitch whose function relies on the ligand-induced formation of a terminator stem (10). The neomycin as well as the tetracycline aptamers were also implemented in ribozymes (11,12). Both aptamers have been also applied to exert translational control through ligand-dependent roadblock formation for the scanning ribosome (5,13). In this mechanism, the regulation relies on the aptamer roadblock stability, rather than the allosteric impact of ligand binding on the aptamer domain-expression platform interface. The ciprofloxacin and paromomycin aptamers have been found to be applicable for translation initiation control via roadblock formation, too (7,8). However, although some new ones have been added recently the specific selection and design of such efficient engineered riboswitches remains a challenge (14).

While the activity of natural riboswitches has specifically evolved in and is therefore adapted to specific cellular conditions and ligand concentrations, the features of *in vitro*-selected aptamers can only be shaped via restrictions in the selection protocol. In general, an exceptionally high affinity seems a pre-requisite for the development of a functional riboswitch. However, high affinity alone is insufficient to predict the activity of those aptamers (15,16). Affinity and several other factors such as the timescale of ligand binding are certainly key elements of riboswitch engineering. However, it is now common consensus that a conformational change due to ligand binding is equally essential to create a functional riboswitch in a cellular environment (8,12–14).

Furthermore, an increased complexity of both sequence and structure (17) is also beneficial and may even enhance the binding affinity (18,19). For neomycin, several aptamers with similar affinities and comparable sizes and structures that mediate different levels of regulation were systematically compared. The results revealed that preformed hairpin structures failed to perform *in vivo*, while other more complex structures with substantial conformational rearrangements were active (5,19). In general, conventional selection protocols often favour the simplest structural solution, i.e. aptamers that do not fold into very complex structures (17). Consequently, there might be a correlation between the increased enrichment of candidates with excellent binding properties, yet poor functionality for the control of gene expression.

**Figure 1.**
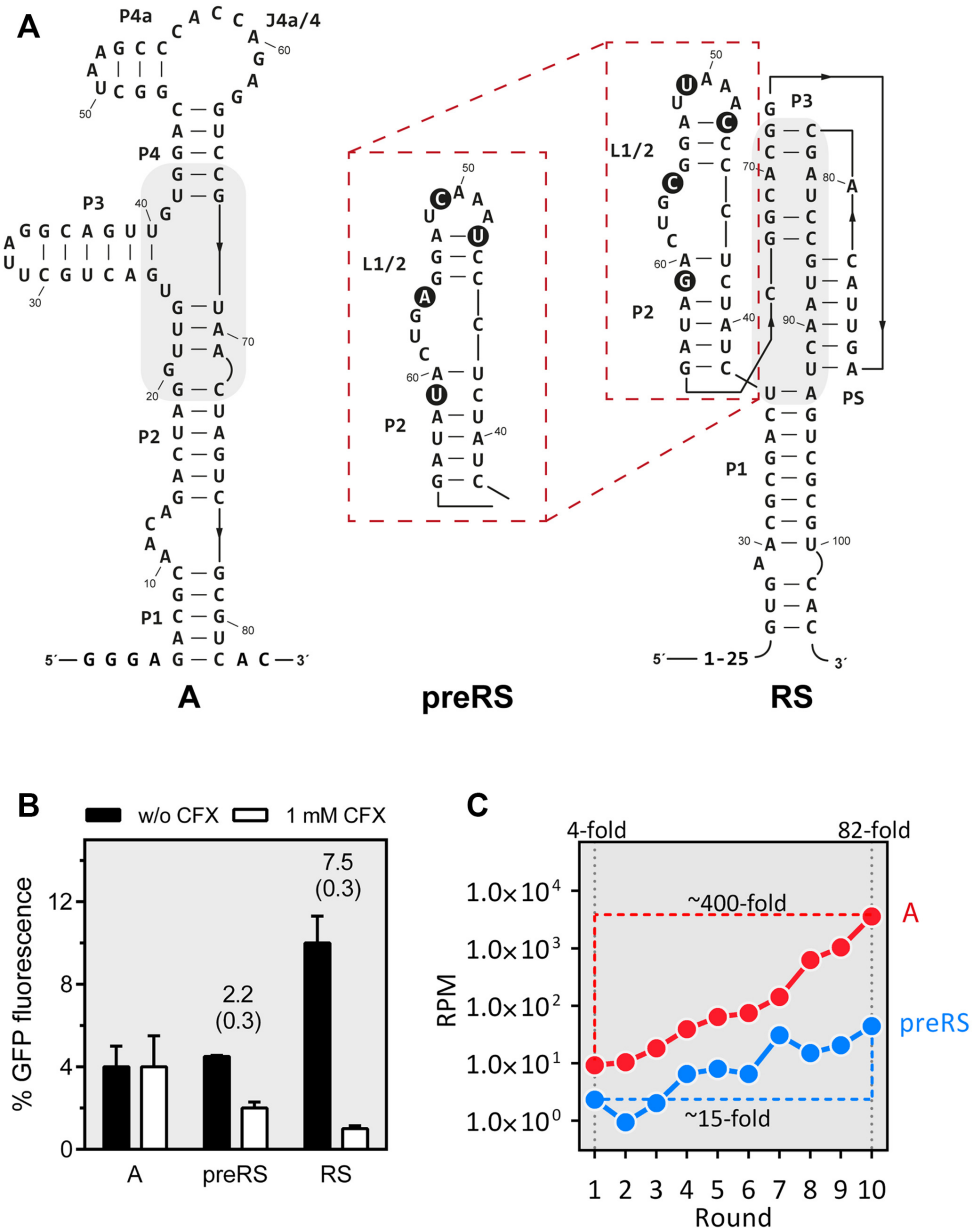
(**A**) Secondary structures of the aptamer **A** and the two riboswitches **preRS** and **RS**, confirmed by *inline* probing (7, 20). Nucleotides that differ between **preRS** and **RS** are highlighted with black circles. Regions involved in ligand binding are shaded in grey. (**B**) Regulation of GFP expression. The aptamer sequences **A**, **preRS** and **RS** are located in the 5′ untranslated region of a GFP reporter gene in *Saccharomyces cerevisiae*. GFP expression is displayed in the absence (black bar) and presence of 1 mM CFX (white bar). The dynamic range of regulation is reported above the bars together with the corresponding errors in parenthesis. (**C**) Deep sequencing data from the SELEX experiment with CFX as target. Reads per million (RPM) are displayed for candidate **A** (red) and **preRS** (blue) that was further developed into **RS** (7).

In essence, a key question of synthetic riboswitch design has yet to be answered in full: what makes an aptamer a good riboswitch? Which factors and characteristics render an aptamer suitable as a high-performance riboswitch for reliable gene expression control? In this study, we explored a parameter potentially affecting the functionality of aptamers as riboswitches that has received little attention to date, the kinetics of the ligand binding process. For this purpose, we compared the ligand binding kinetics of three different aptamers known to bind ciprofloxacin (CFX) (7,20). The three CFX-binding aptamers (Figure 1A) originated from the same SELEX experiment (7). Despite the obvious dissimilarities in their respective sequences and their rather diverse secondary structures, they share a common feature, i.e. their very similar K_D_ values. However, this similarity in affinity does not translate into equal performance, as only two of the candidates were found to be suitable riboswitches. The three aptamers represent the output of three stages in the design process: one was found after ten rounds of selection (candidate **A**), whereas the second was identified via *in vivo* screening of the enriched SELEX pool (candidate **preRS**). Interestingly, deep sequencing analysis (Figure 1C, (7)) revealed that candidate **A** was about 80-fold more abundant in the enriched SELEX pool than **preRS**. The deep sequencing data furthermore showed that the enrichment trajectory for R10K6 (precursor of **A**) was significantly steeper than for **preRS**, thus demonstrating that the selection protocol clearly favours R10K6. Finally, the third construct **RS** was obtained by distinct mutations of single nucleobases close to the binding pocket region of preRS to optimize activity (Figure 1B). In each of the investigated aptamers, the inherent fluorescence of the ligand is equally quenched upon binding which provides a convenient spectroscopic read-out for ligand binding studies. Our results for the binding kinetics and the Mg^2+^ dependence of the aptamers described above revealed key differences that we assume to be critical for riboswitch engineering of *in vitro* selected aptamers.

## MATERIALS AND METHODS

### Preparation of RNA aptamers

For *in vitro* analysis, RNA was transcribed from PCR-generated templates, all containing at least one 5′-terminal guanosyl residue to facilitate *in vitro* transcription using T7 RNA polymerase. For this, two oligonucleotides were designed with an overlap of 30 bp (CCAAGTAATACGACTCACTATAGGGAGACGCAACAGACTAGGTTGTGACTGCTTAGGCAGTTGTGGACGG and GTGACGCGACTAGTTACGGACCTCTGGTGGGCTTAGCCGTCCACAACTGCCTAAGCAGTCACAACCTAGTC for **A**, CCAAGTAATACGACTCACTATAGGGAGACGCAACTGAATGAACATAAGTGAACGCGACTCTATCTCCCTAAACTAGG and GTGACGCGACTAGTTACGGATCGTGTAACTCCGTGCCGCTATATGACTCCTAGTTTAGGGAGATAGAGTCGCGTTC for **preRS** and CCAAGTAATACGACTCACTATAGGGAGACGCACCTGAATCAACATACGTGAACGCGACTCTATCTCCCCAAATTAGGCGTCAG and GTGACGCGACTAGTTACGGATCGTGTAACTCCGTGCCGCTATCTGACGCCTAATTTGGGGAGATAGAGTCGCGTTCACG for **RS**) and amplified using Q5® High-Fidelity DNA polymerase (NEB) according to the supplier's instructions. After ethanol precipitation, the DNA template was used for *in vitro* transcription by T7 RNA polymerase (NEB) as reported previously (7). The RNA was gel purified (21) and molarity was determined by spectrophotometric measurement using NanoDrop 1000 Spectrophotometer (Thermo Scientific).

The RNA samples were stored in highly purified water at −20°C. Prior to each experimental use, the RNA was prepared using the following folding procedure: the aqueous RNA solutions were heated to 95°C for 5 min and snap-cooled on ice for 5 min. Next, buffer was added to a final concentration of 40 mM HEPES, 125 mM KCl, 5 mM MgCl_2_, pH 7.4 and equilibrated for 20 min. The composition of this buffer is equivalent to the SELEX buffer.

### Fluorescence titration experiments

Dissociation constants (*K*_D_) for CFX@RNA complexes were determined by measuring the fluorescence quenching as a function of RNA concentration in the presence of a fixed CFX concentration of 50 nM. Fluorescence intensities were measured on a Fluorolog FL3-22 (Horiba Jobin Yvon) with an excitation wavelength set to 335 nm (slit 5 nm) and an emission wavelength of 420 nm (slit 5 nm). The integration time was set to 0.5 sec and temperature was adjusted to 25°C. In between the addition of RNA, the solution was stirred for 1 min and equilibrated for an extra minute. For the titration experiments, 50 nM CFX in 40 mM Hepes, 125 mM KCl, 5 mM MgCl_2_, pH 7.4 (F_0_) was mixed with increasing amounts of gel-purified and folded RNA (see above: Preparation of RNA aptamers) and fluorescence intensity was measured (F). Curve fitting was done using Prism (GraphPad Software) and non-linear regression analysis with the modified Hill equation (1) by least squares fitting:

(1) $$\begin{equation*}\frac{{\boldsymbol{F}}}{{{{\boldsymbol{F}}_0}}} = {\boldsymbol{\ }}{{\boldsymbol{B}}_{{\boldsymbol{{\rm max}}}}}\frac{{{{\boldsymbol{X}}^{\boldsymbol{h}}}}}{{\left( {{{\boldsymbol{K}}_{\boldsymbol{{\rm D}}}}^{\boldsymbol{h}} + {{\boldsymbol{X}}^{\boldsymbol{h}}}} \right)}}\end{equation*}$$

with *B*_max_ = maximum binding, *h* = hill slope, *X* = concentration of RNA.

### Time-correlated single photon counting

Spectroscopic measurements of fluorescence decay of the ligand CFX and the ligand-aptamer complexes were carried out in the same buffer as used in the SELEX procedure and the fluorescence titrations. The experiments were performed with a self-assembled time-correlated single photon counting (TCSPC) setup incorporating a single-photon detection photomultiplier tube (PMT, PMA-C 182 M, PicoQuant, Berlin, Germany) and a TimeHarp 260 PICO Single PCIe card (PicoQuant) for data processing (22). Pulsed orthogonal excitation of the samples was achieved with a pulsed LED PLS310 (PicoQuant). Multi-exponential fitting was performed with FluoFit Pro 4.6 [PicoQuant, (23)]. The samples were measured in 4 × 10 mm quartz glass cuvettes at concentrations of 1 μM and 2 μM for CFX and RNA aptamers, respectively.

### Stopped-flow spectroscopy

Stopped-flow measurements were carried out with a SFM-20 device (Bio-Logic Science Instruments, Seyssinet-Pariset, France) with Berger Ball mixer and a cuvette (FC08) with a volume of 20 μl and a light path of 0.8 mm attached. The stopped-flow device was coupled to a FP-8500 spectrofluorometer (Jasco, Groß-Umstadt, Germany) with a glass-fibre module (OBF-832, Jasco). The detected PMT signal was transferred to a transient recorder board (PCI-6052E, National Instruments, Austin, USA) using an A/D-adapter (BNC-2110, National Instruments). Data acquisition was controlled with the Bio-Kine 32 software (Version 4.42, Bio-Logic Science Instruments). For every single mixing experiment, 33 ml of the two sample solutions were injected into the mixing compartment via syringes (Hamilton 1010C, Hamilton Company, Reno, USA) with a flow rate of 6.95 ml/s. The injection was stopped by a hard-stop valve which determined the start of the observed binding dynamics. The excitation wavelength for the measurements was 330 nm and fluorescence emission was detected at 420 nm and a 90° angle. The applied RNA concentration was 2 μM and the CFX concentrations were 4, 8, 12, 16 and 20 μM, both in 40 mM HEPES, 125 mM KCl, 5 mM MgCl_2_, pH 7.4. The RNA was folded before use as described above (see Preparation of RNA aptamers). 20–30 traces were averaged for each concentration. All of the traces were baseline-corrected and normalized prior to the kinetic model analysis, which was carried out with the DynaFit4 software (24) (Biokin Ltd, Watertown, USA). A detailed description of the raw data processing routine (Supplementary Figure S2) and subsequent analysis (Supplementary Tables S2–S7 and Supplementary Figures S3–S5) may be found in the Supporting Information (SI).

## RESULTS

### Mg^2+^ dependence of ligand binding

Despite the relatively low chemical diversity compared to proteins, the conformational space of RNA is enormous. The distribution of defined structures can be greatly altered by interactions with substrate molecules or ions. There may even be novel structures that would not be stable without those interactions (25). Cations, especially divalent Mg^2+^ ions, are essential in stabilizing tertiary RNA structures and are thus required for most aptamers to fold into their native and binding-competent state. However, it is suggested that excess availability of divalent cations favours the accumulation of aptamers with poor structural complexity. Generally, more complex aptamer structures are assumed to undergo more profound conformational changes upon ligand binding (17). Although SELEX is often carried out at high ionic concentration conditions (5–10 mM Mg^2+^) to obtain well-binding aptamers, synthetic riboswitches have to be able to cope with lower Mg^2+^ concentrations (around 1.5 mM) to remain functional in physiological conditions. Therefore, our first approach was to investigate whether the Mg^2+^ concentration had an influence on the ligand binding of our aptamer candidates in the relevant concentration range of Mg^2+^.

**Figure 2.**
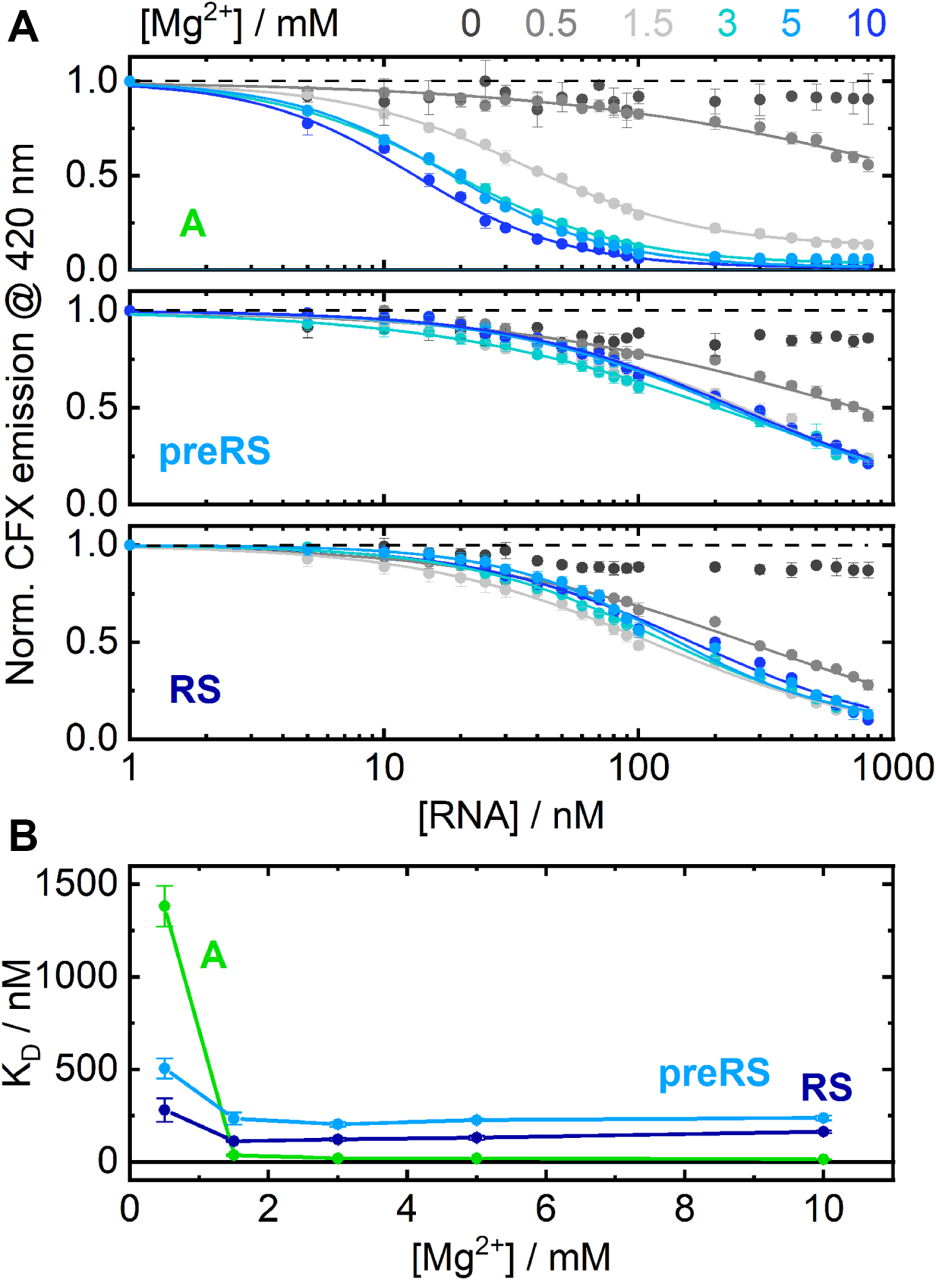
(**A**) RNA-titration series, showing the detected CFX emission at constant Mg^2+^ concentrations for candidate **A** (upper panel), **preRS** (middle panel) and **RS** (lower panel). (**B**) Calculated K_D_ values plotted as a function of the applied Mg^2+^ concentrations. Candidate **A** is shown in green, **preRS** in light blue and **RS** in dark blue.

We conducted fluorescence titration experiments at defined Mg^2+^ concentrations (Figure 2A). Comparison of the corresponding apparent *K*_D_ values (Figure 2B) revealed differences in RNA behaviour. Aptamer **A** showed a clear cation dependence at low Mg^2+^ concentrations. In the absence of divalent cations, no ligand binding to aptamer **A** was observed at all. At 0.5 mM Mg^2+^, a drop in fluorescence of almost 50% was detected. Evidently, small amounts of Mg^2+^ are sufficient to enrich preformed structures and induce ligand binding to a considerable extent. From a concentration of 1.5 mM Mg^2+^, the detected emission already decreased to a residual level. The conformational distribution was therefore mostly shifted towards binding-competent structures. The corresponding apparent *K*_D_ values were all in the low nanomolar range with no significant improvements upon further increase of the Mg^2+^ concentration. They reach a minimal *K*_D_ value of ∼13 nM at 10 mM Mg^2+^.

For the candidates **preRS** and **RS**, no evident Mg^2+^ dependence could be observed. At 0.5 mM Mg^2+^, the conformation shift of candidate **preRS** towards a binding-competent state seemed to be already mostly complete, as implied by a total fluorescence drop of >50%. Finally, very similar binding curves from a Mg^2+^ concentration of 1.5 mM upwards were detected for both active candidates. These experiments show that there are differences in ligand binding depending on the concentration of Mg^2+^, but only at very low concentrations. No changes were observed at physiological concentration (1.5 mM) or higher (concentration used for the SELEX). Consequently, we decided to retain a concentration of 5 mM for all subsequent experiments to mimic SELEX conditions.

### Fluorescence quenching

**Figure 3.**
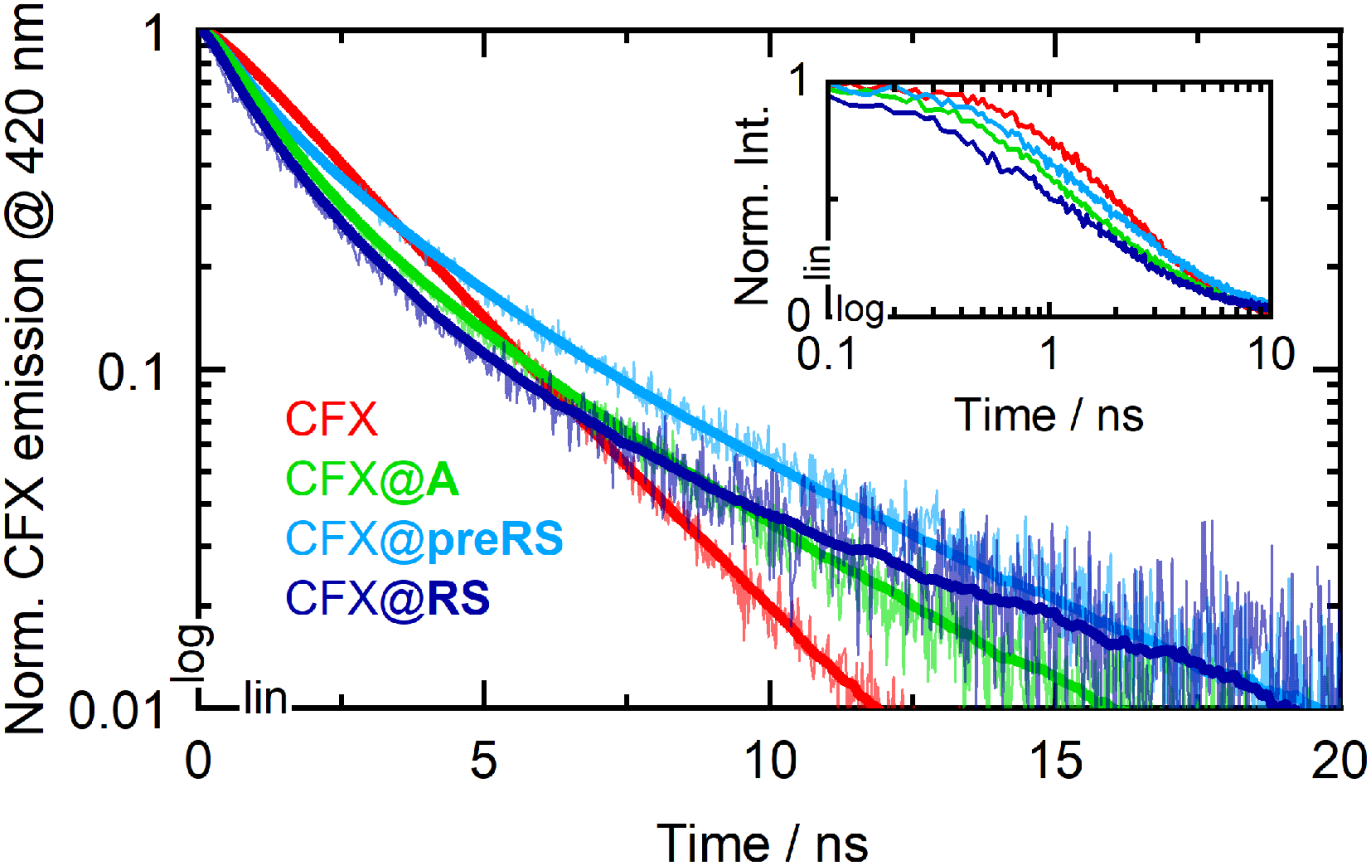
Fluorescence decays of CFX (red) and the ligand-bound complexes CFX@**A** (green), CFX@**preRS** (light blue) and CFX@**RS** (dark blue). The solid bold lines represent the fitted curves, yet they are omitted in the inset for better data visibility.

In a next step, we measured the fluorescence quenching effects upon binding. Fluorescence decays of CFX and the CFX@RNA complexes were recorded with time-correlated single photon counting (TCSPC) experiments (Figure 3). The datasets were subsequently analyzed by multi-exponential data fitting to determine the corresponding fluorescence lifetime contributions. The ligand CFX was measured in different buffer compositions because fluoroquinolones are known to form various types of metal complexes, which might influence the photophysical properties (26). The respective data and a description of the fitting routine may be found in the SI (see Supplementary Figure S1 and Supplementary Table S1). Most prominently, CFX bears a 1,3-dicarbonyl functional group that enables the formation of dimers like [Mg(CFX)_2_]. The carbon acid or the piperazine moiety might also be able to form metal chelates. Without cations present, a mono-exponential decay with a lifetime of 1.3 ns was observed. Upon addition of potassium or Mg^2+^, biexponential decays were recorded with minor lifetime components around 6 ns. The faster components slightly differ from the 1.3 ns lifetime, supposedly due to the multiple coordination sites for metal ions and for water molecules. Moreover, CFX exhibits several protonation sites with p*K*_a_ values in the neutral regime, which can also have a significant impact on the fluorescence features (27,28).

Under buffer conditions applied during the SELEX, i.e. containing potassium as well as Mg^2+^, the fluorescence decay of CFX was modelled biexponentially with a main time constant **τ**_1_ = 1.3 ns, accounting for the pure ligand (Table 1). A minor and slower component **τ**_2_ = 2.9 ns was assigned to the decay of chelate complexes mainly formed with Mg^2+^. The average lifetime of CFX under these buffer conditions was determined to be 1.9 ns. For the CFX@RNA measurements, an additional sub-ns component was required for adequate fitting of the decay curves. This time constant has the largest amplitude of the corresponding decay curves and thus reflects the accelerated fluorescence emission due to quenching (**τ**_q_) within the CFX@RNA complexes for all three candidates. For aptamer **A**, the contribution of **τ**_q_ = 0.64 ns was the largest with 77%. The quenching lifetimes of the candidates **preRS** and **RS** were 0.43 ns and 0.49 ns, respectively, and showed significantly lower amplitudes.

**Table 1.** Fluorescence lifetimes **τ** and their standard error values determined by multi-exponential curve-fitting with corresponding amplitudes A in percent provided in parentheses together with their relative errors.

	τ_q_^[a]^ / ns (*A*_q_ / %)	τ_1_^[b]^ / ns (*A*_1_ / %)	τ_2_^[b]^ / ns (*A*_2_ / %)	χ^2^
CFX	–	1.28±0.02 (65±2)	2.92±0.04 (35±2)	1.003
CFX@**A**	0.64±0.03 (77±3)	2.48±0.05 (14±5)	6.5±0.3 (9±5)	0.991
CFX@**preRS**	0.43±0.03 (53±5)	2.22±0.04 (37±2)	6.3±0.2 (10±3)	1.034
CFX@**RS**	0.49±0.03 (58±5)	1.90±0.06 (36±3)	8.3±0.5 (6±5)	1.051

^[a]^ **τ_q_** represents the fluorescence quenching lifetimes of the CFX@RNA complexes, ^[b]^**τ_i_** represent the remaining consecutively numbered lifetime components.

These lower amplitudes are in line with the higher binding affinity of **A** in the given experimental conditions. The time constants **τ**_1_ are in the range of 1.9–2.5 ns for the CFX@RNA measurements. They correspond to the residual unbound ligand that still fluoresces strongly and its resulting metal and hydrate complexes formed in aqueous solution. The lifetimes **τ**_1_ found in the **preRS** and **RS** measurements are well in agreement with the average lifetime of the ligand CFX in buffered solution and their amplitudes are higher by a factor of 2.6 than that of the corresponding lifetime of CFX@**A**. The minor lifetime components **τ**_2_ are clearly slowed down in each case and show amplitudes smaller than 10%. As lifetimes of roughly 6 ns were also found for the ligand in different buffer conditions, those lifetimes could again be assigned to particular chelate complexes or protonated states of CFX. Furthermore, the respective lifetimes might also account for discriminative aptamer-ligand complexes where the ligand encounters a different aptamer conformation, which may in turn affect emission properties. Although it is not clear whether the binding of CFX is stabilized via e.g. H-bonds or stacking interactions, the similar fluorescence quenching lifetimes suggest similar microenvironments for the ligand embedded in the binding pocket of **preRS** and **RS** compared to **A**. Possible quenching mechanisms are typically divided into static and dynamic quenching. Static quenching refers to the formation of an aptamer-ligand complex with altered ground state properties, so that the population of the original fluorescent state of the pure ligand is inhibited (29). Since the absorption spectrum of the ligand does not change upon addition of RNA and residual fluorescence at the same wavelength is detected for the free ligand as well as for the bound states, static quenching is unlikely to be the main mechanism. However, dynamic quenching refers to any interaction of the aptamer with the excited state of the fluorophore. As an accelerated fluorescence lifetime of the CFX@RNA complexes was detected, we assume that the emission of CFX is quenched dynamically in the excited state of the complexes.

### Fluorescence-monitored binding kinetics

**Figure 4.**
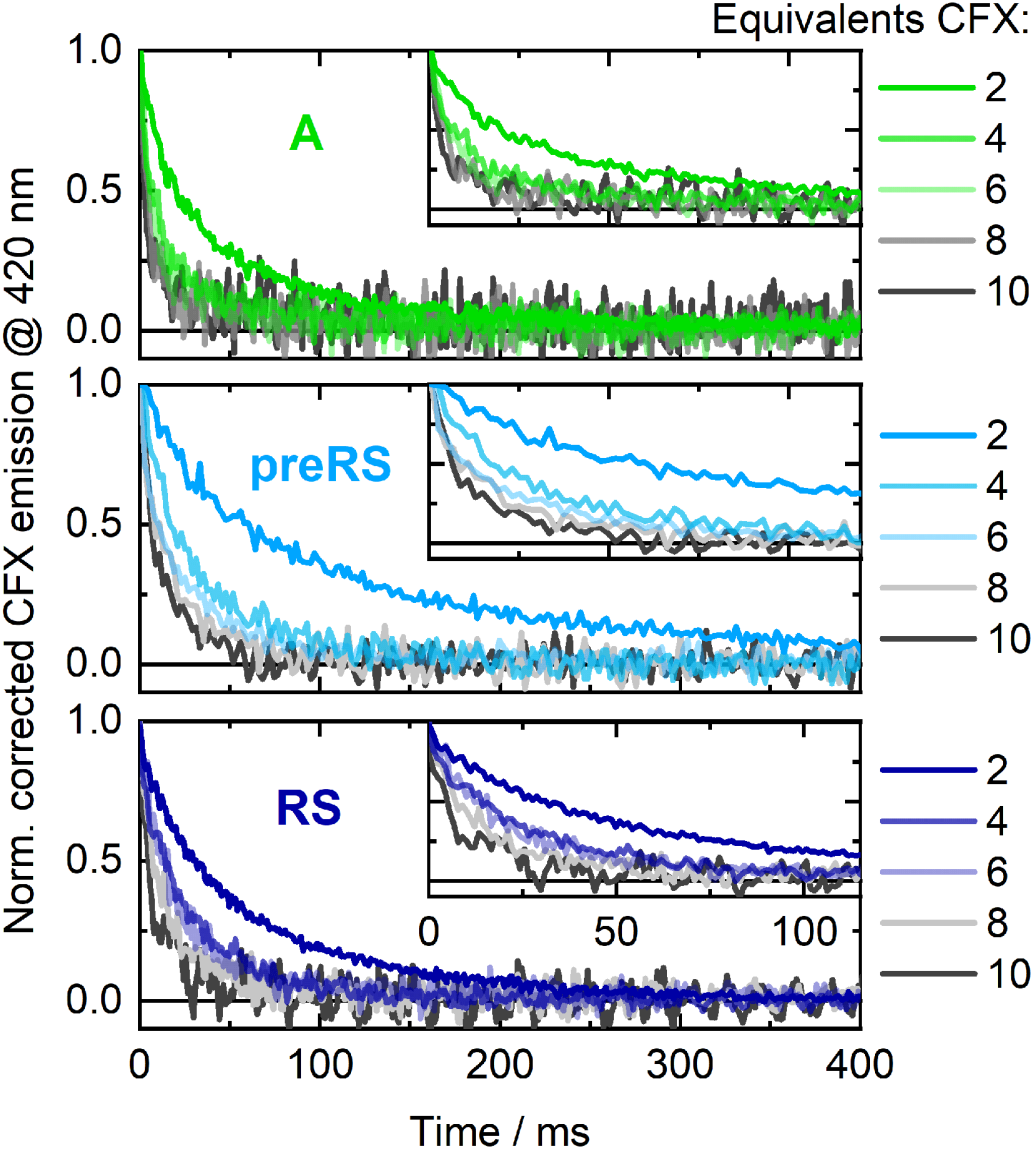
Fluorescence-detected stopped-flow measurements of the CFX-binding dynamics of the aptamer **A** (upper panel) and the riboswitches **preRS** (middle panel) and **RS** (lower panel) upon mixing the RNAs with different concentrations of ligand (relative equivalents indicated).

In the stopped-flow experiments, the decrease of CFX emission caused by aptamer binding was monitored in a time-resolved manner on the milliseconds timescale. The ligand CFX was supplied in excess relative to the specific RNAs (2, 4, 6, 8 and 10 equivalents = eq), which resulted in pseudo-first order complex formation. Due to the saturation of the particular RNAs with supplied ligand, a similar signal amplitude was obtained for each measurement series. The data were recorded under similar conditions as in the SELEX procedure. Hence, both the ligand CFX and the candidate RNAs were supplied in the same buffer conditions in the separate syringes of the stopped-flow apparatus, so that the buffer conditions were maintained upon mixing of components. RNA was properly folded prior to usage as described. At the applied Mg^2+^ concentration (5 mM), the ensemble of present RNA structures was expected be mostly shifted towards the preformed state for each of the candidates, as shown by the Mg^2+^-dependent titrations. The observed kinetics should therefore merely reflect the ligand binding event of binding-competent RNA conformations and no superimposed conformational dynamics should impede data analysis. The processed and averaged time-dependent traces of the CFX emission during ligand binding are illustrated in Figure 4.

**Figure 5.**
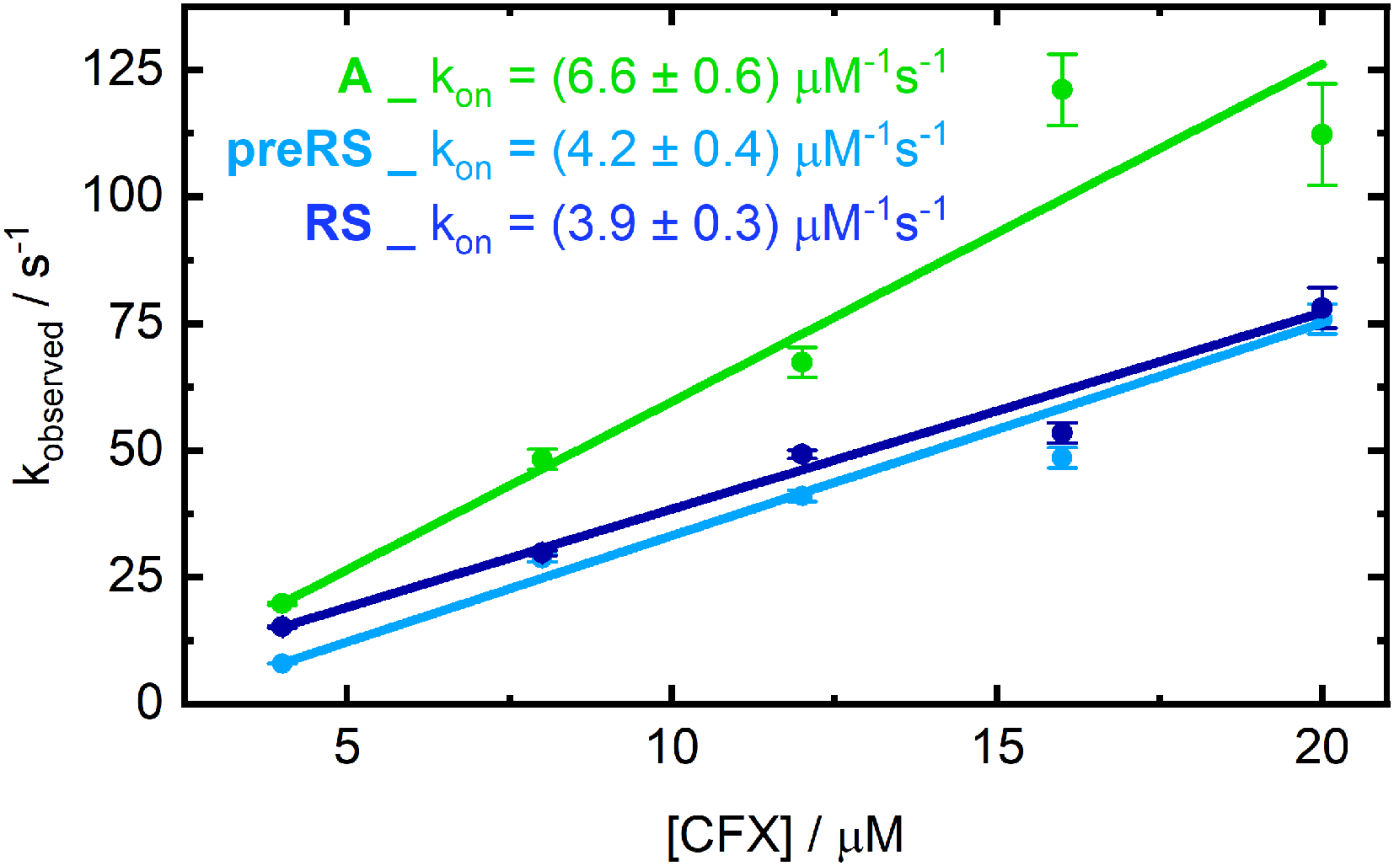
Observed first order association rates obtained from mono-exponential fitting of the stopped-flow traces plotted against the ligand concentration. The slope of the linear fits corresponds to the overall rate *k*_on_.

In each case, binding is clearly accelerated with increasing amounts of ligand added. In essence, the observed dynamics were significantly faster for the aptamer **A** than for both potential riboswitches. For the 2 eq curves, the equilibrium level of the aptamer **A** was already reached after roughly 150 ms, whereas it took ∼300 ms for **RS** and 400 ms for **preRS**. Moreover, the acceleration of the dynamics of **A** is almost at its limit with 6 eq of CFX, whereas the dynamics become continuously faster for higher amounts of supplied ligand for the potential riboswitches. Under pseudo-first order conditions, fitting the obtained stopped-flow transients monoexponentially allows for an estimation of the observed association rate for each ligand concentration. Plotted against the corresponding CFX concentration, this reveals the apparent *k*_on_ through linear regression (Figure 5).

Although a simple irreversible one-step binding model is clearly just an approximation of the actual kinetics, a monotonous increase of the association rate is evident. This indicates an induced fit mechanism of already pre-existing binding-competent aptamer structures (30) and confirms the preformation of the RNA candidates in the given conditions. However, there are no firm criteria to exclude the contribution of conformational selection (31). The existence of multiple active or inactive conformations cannot be ruled out, especially if the equilibration between those structures is even faster than the ligand binding event and thus not rate-limiting. For our data, the association rate *k*_on_ reached a plateau at ligand CFX concentrations higher than 10 equivalents relative to the applied RNA, which could indicate such a limitation by conformational selection in this ligand concentration range. A comparable behaviour was already observed for other *in vitro* selected aptamers like the theophylline-binding aptamer (32), whose apparent *k*_on_ is one order of magnitude smaller than the rates determined here. For natural riboswitches, e.g. the purine riboswitch (33), a rate-limiting conformational selection was also reported.

For more detailed analyses, the obtained and processed datasets were fitted globally (i.e. each time trace of the particular concentration series simultaneously) to multiple simple and meaningful kinetic models including different combinations of reversible and irreversible binding steps. The tested models are listed in Table 2 and the datasets were fitted with the corresponding sets of differential equations resulting from the reaction mechanisms. A detailed explanation of the fitting routine of the processed data and the particular differential equations may be found in the SI, complete with the obtained fit results (Supplementary Tables S2–S7) and the fitted curves (Supplementary Figures S3–S5).

**Table 2.** Reaction schemes of tested binding models.

Model	Reaction scheme
1	${\rm RNA} + {\rm CFX} \mathop \rightleftharpoons \limits^{\rm k_{1}}_{\rm k_{-1}} {\rm CFX}@{\rm RNA}$
2	${\rm RNA{_{\rm inactive}}^{\rm [a]}}\mathop \rightleftharpoons \limits^{\rm k_{1}}_{\rm k_{-1}} {\rm RNA{_{\rm active}}^{\rm [b]}}+{\rm CFX} \mathop \rightleftharpoons \limits^{\rm k_{2}}_{\rm k_{-2}} {\rm CFX}@{\rm RNA}_{\rm active}$
3	${\rm{RNA\ }} + {\rm{\ CFX\ }}\mathop \to \limits^{{{\rm{k}}_1}} {\rm{\ CFX}}@{\rm{RNA^{*}\ }^{[c]}}\mathop \to \limits^{{{\rm{k}}_2}} {\rm{\ CFX}}@{\rm{RNA}}$
4	${\rm RNA}+{\rm CFX}\mathop \rightleftharpoons \limits^{\rm k_{1}}_{\rm k_{-1}} {\rm CFX}@{\rm RNA}^*\mathop \rightleftharpoons \limits^{\rm k_{2}}_{\rm k_{-2}} {\rm CFX}@{\rm RNA}$
5	${\rm RNA}+{\rm CFX}\mathop \rightleftharpoons \limits^{\rm k_{1}}_{\rm k_{-1}} {\rm CFX}@{\rm RNA}^*\mathop \to \limits^{{{\rm{k}}_2}} {\rm CFX}@{\rm RNA}$

^[a]^ RNA_inactive_ – non-binding state of the aptamer, ^[b]^ RNA_active_ – binding-competent state of the aptamer, ^[c]^ CFX@RNA* – intermediate bound state.

Comparison of the respective fit qualities by means of the Akaike information criterion (AIC) and the Bayesian information criterion (BIC) allowed an evaluation of the applied models (24). Both criteria estimate relative fit qualities considering both the number of free parameters included in a particular statistical model and its accuracy (root mean square deviation, RMSD) to avoid over-parametrization. Compared to the AIC, the BIC penalizes the number of parameters more severely. For model assessment, the differences ΔAIC and ΔBIC of the compared models were calculated.

In the simplest model 1, a reversible one-step binding was assumed. In contrast to the mono-exponential fitting shown before, this model takes the back reaction into account and therefore allows for an estimation of the apparent dissociation rate *k*_-1_. The determined *k*_1_ values, based on a reversible one-step binding, are in agreement to the *k*_on_ values obtained from mono-exponential fitting (Table 3). The obtained fit qualities were the poorest of all, which is reflected by the highest values for ΔAIC and ΔBIC. However, the estimated back rates *k*_-1_ considerably decrease from **A** to **preRS** to **RS**. For the active candidate **RS**, a meaningful value for *k*_-1_ could not be determined because the respective rate constant approached zero. The dissociation rate of **RS** is therefore almost negligible and the reduced *k*_-1_ values of **preRS** and **RS** seem to correlate with their increased *in vivo* activities.

**Table 3.** Rate constants determined from kinetic analysis for the reversible one-step model 1. The ΔAIC and ΔBIC criteria show the fit accuracies compared to the other models.

	*k*_1_ / μM^−1^s^−1^	*k*_-1_ / s^−1^	RMSD^[a]^	ΔAIC^[b]^	ΔBIC^[c]^
**A**	7.78±0.09	0.33±0.03	0.044783	801	788
**preRS**	3.61±0.03	0.09±0.01	0.030391	742	733
**RS**	5.21±0.04	<10^−6^	0.03298	701	694

^[a]^ Obtained root mean square deviation values, ^[b]^ Akaike information criteria differences to the most accurate model, ^[c]^ Bayesian information criterion differences to the most accurate model.

Conformational selection (model 2) should play only a minor role under the given experimental conditions. The reaction scheme assumes a preformation of the aptamer from an inactive to an active binding-competent conformation, which then undergoes ligand binding in one reversible step. Indeed, application of model 2 did not result in a sufficient goodness of fit for any of the RNA candidates. The obtained rates of the ligand binding step were slightly higher than the association rates from model 1 and the preformation occurs on a similar timescale for **A** and **preRS**. Interestingly, the prefolding rate of the candidate **RS** is determined to be 8 times higher than those of the other two candidates. The two irreversible steps assumed in model 3 would be relevant if two highly favoured structural rearrangements occur sequentially. Such a behaviour was e.g. shown for the aptamer domain of the guanine-sensing riboswitch (34). However, model 3 also failed as the model of choice for aptamers explored here, according to the obtained fit criteria.

The most suitable two-step models for the three candidates and the determined rates and fit quality criteria are summarized in Table 4. For the binding kinetics of the aptamer **A**, the reversible two-step model 4 is the most adequate description, as it scores the lowest RMSD and both likelihood criteria ΔAIC and ΔBIC are zero. The analysis revealed the highest bimolecular association rate (*k*_1_ = 10 μM^−1^s^−1^) and the lowest back-rate (*k*_-1_ = 1.6 s^−1^) of the first binding step of all candidates. This step is considered as an initial association of the ligand to the binding site of the RNAs, presumably driven by unspecific electrostatic interactions or even hydrogen bonding. Starting from the intermediate complex CFX@RNA*, the formation of the final ligand-bound of **A** state is favoured because the rate (*k*_2_ = 4 s^−1^) is 2.5 times higher than the dissociation rate *k*_-1_. The second step accounts for a tightening of the binding pocket, accompanied by the establishment of specific interactions with CFX. The reverse rate of the second step (*k*_-2_ = 2.3 s^−1^) is significantly lower than the forward rate but in the same order of magnitude. Similar to aptamer **A**, application of model 4 also yielded the lowest RMSD and ΔAIC for **preRS**, although the determined rates are quite different. The initial association step is clearly less favoured (*k*_1_ = 4.33 μM^−1^s^−1^) and the dissociation rate is increased (*k*_-1_ = 5.7 s^−1^). Furthermore, the back-rate of the second step (*k*_-2_ = 0.06 s^−1^) is almost negligible as it is substantially smaller than the forward rate (*k*_2_ = 13.2 s^−1^). The *k*_2_/*k*_-2_ ratio for aptamer **A** is only 1.8, but for **preRS** it is as high as 220. Although model 4 scores a slightly lower RMSD than model 5 for **preRS**, the latter is preferred because of the greater simplicity of the model, which is indicated by its lower ΔBIC value. The determined rates are all slightly lower than those obtained from model 4. Consequently, the second binding step of **preRS** is not only much faster than in the case of aptamer **A**, but it is virtually irreversible. For the final construct **RS**, model 4 achieves very good fit values, but model 5 is clearly the best approximation according to its lowest RMSD value and likelihood criteria. The determined association rate of **RS** (*k*_1_ = 7.6 μM^−1^s^−1^) is almost two times higher than that of **preRS**. Additionally, the dissociation rate is lower (*k*_-1_ = 3.1 s^−1^), so the initial binding of **RS** proceeds much faster. The second binding step of **RS** exhibits the highest rate (*k*_2_ = 23 s^−1^) among all investigated RNAs. Compared to **preRS**, it is accelerated by almost a factor of two and compared to **A**, it is even faster. Hence, there is a clear trend towards an accelerated conformational adaption that seems to correlate with the increasing regulatory potential of the three RNA candidates.

**Table 4.** Rate constants determined from kinetic analysis for the most suitable two-step binding models 4 and 5 and RMSD values of the corresponding fits. The ΔAIC and ΔBIC criteria show the fit accuracies compared to the other models.

Model		*k*_1_ / (μM s)^−1^	*k*_-1_ / s^−1^	*k*_2_ / s^−1^	*k*_-2_ / s^−1^	RMSD^[a]^	ΔAIC^[b]^	ΔBIC^[c]^
**A**	4	10±0.2	1.6±0.2	4±0.2	2.3±0.2	0.041255	0	0
	5	10±0.2	0.8±0.2	6±0.3	—	0.041582	75	69
**preRS**	4	4.33±0.06	5.7±0.5	13.2±0.8	0.06±0.03	0.028187	0	5
	5	4.31±0.06	5.4±0.4	12.1±0.7	—	0.028198	2	0
**RS**	4	7.6±0.1	3.1±0.6	23±1	0.27±0.05	0.030741	4	10
	5	7.62±0.11	2.9±0.7	23±1	—	0.030736	0	0

^[a]^ Obtained root mean square deviation values, ^[b]^ Akaike information criteria differences to the most accurate model, ^[c]^ Bayesian information criterion differences to the most accurate model.

Interestingly, the obtained reaction rates of the initial step substantially differ between the potentials riboswitches and the inactive aptamer. The association of CFX with Aptamer **A** is significantly favoured over CFX association with the riboswitches, which indicates profound differences in the degree of preformation and the interactions established upon first encounter. In all likelihood, aptamer **A** exists in a rather compact conformation, due to the increased number of incorporated Mg^2+^ ions and is thus highly preformed. The initial binding to the potential riboswitches is supposedly less specific and the binding pocket less structured. However, the essential difference of the binding kinetics is the intense acceleration of the second step. The negligible rate *k*_-2_ renders the respective step virtually irreversible for **preRS** and **RS** during the recorded association phase.

We assume that for the riboswitch candidates, the second binding step corresponds to a highly favoured structural adaptation of the RNA that results in a tightening of the binding pocket. Put another way, a ‘nearly’ irreversible final binding step could indicate a structural rearrangement so substantial that it can mediate riboswitch activity. In addition, a distinct correlation between the reduction of the respective back-reaction rate and an increasing riboswitch activity in the order from candidate **A** to **preRS** to **RS** is observed.

## DISCUSSION

Previous studies suggest that the overall association rate and the aptamer-ligand complex lifetime are essential characteristics to describe riboswitches, rather than the *K*_D_ value (16). Moreover, significant differences between kinetically derived *K*_D_ values and equilibrium values of *in vitro* selected aptamers are observed quite often (35). Such differences are of particular importance since *in vivo* riboswitch activity can generally rely on kinetic or thermodynamic control, which is apparently unknown for *de novo* engineered riboswitches. For kinetically driven riboswitches, the amount of ligand necessary for significant regulation is typically higher than the *K*_D_ derived estimate (36). The regulation mechanism for such riboswitches relies on the competition of ligand binding and riboswitch folding. However, the selection protocol prefers enrichment of thermodynamically controlled aptamers where the ligand-bound state is energetically favoured.

Binding kinetics similar to those observed here, i.e. an almost irreversible final binding step that accounts for a structural adaptation of the RNA as for candidate **RS**, were reported for the tetracycline-binding aptamer. The forward rate of the first step is only slightly faster as determined for candidate **RS**, but the back rate of the tetracycline-binding aptamer is drastically higher by a factor of almost 12. Moreover, the rate of the second irreversible step is as high as 155 s^−1^ (37). This step is supposed to rely on a ligand-induced structural adjustment of the aptamer (38,39). A reversible two-step binding mechanism has also been found to govern the neomycin-binding aptamer (N1), where the back rate of the second step is significantly reduced, relative to the forward rate (40). Additionally, a significant conformational adaption was demonstrated for the ligand binding of the N1 aptamer that seems to be vital for its *in vivo* activity. Among the multiple neomycin-binding aptamers that were obtained via *in vitro* selection, other candidates with similar nanomolar *K*_D_ values but different riboswitching capabilities were identified by parallel *in vivo* screening (41). By comparing the NMR structures of the free and the ligand-bound states of three of those aptamers, a clear correlation between their structural complexity and the degree of preformation and conformational switching upon binding was shown (5). Consistently, the greatest regulatory potential is reported for the N1 aptamer as it exhibits the most profound structural change. For the theophylline aptamer, a similar linear increase of the apparent *k*_on_ was observed at low ligand concentrations in the micromolar range. However, a conformational preformation was identified as the rate-limiting step at millimolar theophylline concentrations and a structural adaption was shown to occur upon ligand binding, which renders the aptamer functional *in vivo* (32). By comparing two selected streptomycin-binding aptamers with varying ligand specificities, the conformational change upon ligand binding was even assigned as the driving force for ligand discrimination (42). Furthermore, a significant stabilization of the streptomycin aptamer structure due to ligand binding is reported, indicating an energetically favoured complex formation (43).

Analogous binding kinetics have been reported for the aptamer domain of the purine riboswitch, thus indicating that this binding behaviour is not limited to synthetic riboswitches, but can also be observed in their natural counterparts (33). A slow interconversion of an ensemble of disordered RNA structures towards one ligand binding state is preceding a highly favoured conformational change of the binding pocket to enclose the ligand. The same is true for the guanine-binding riboswitch, where the accommodation of the ligand can even be described by two consecutive irreversible steps to form the final complex (34). For the thiamine pyrophosphate (TPP) riboswitch, a pronounced ligand-induced structural adaption was confirmed by means of fluorescence stopped-flow experiments with 2-aminopurine labelled RNA variants (44). Moreover, kinetic analyses of ITC data revealed the dynamics of the two-step binding sequence, with a rapid initial association of TPP to the aptamer domain followed by the adjustment of the aptamer conformation. The back rate of the second step was determined to be several orders of magnitude smaller than the forward rate, indicating a virtually irreversible ligand induced folding (45). Other examples with similar trends are the adenine-sensing riboswitch (16) or the flavin mononucleotide (FMN) riboswitch (46). Therefore, it is plausible that the underlying ligand binding kinetics beyond the binding constant also dictate the regulatory potential of RNA aptamers. An irreversible second binding step apparently accounts for a highly favoured structural adjustment and extends the lifetime of the ligand-aptamer-complex, which seems to be a prerequisite for its functionality as a riboswitch. Furthermore, examples of similar binding kinetics are found for many possible regulatory mechanisms in both natural and engineered riboswitches. For some mechanisms of riboswitches, it is essential that ligand binding induces a structural change of the binding pocket that also affects the conformation of the expression platform. The formation of a terminator stem adjacent to the aptamer domain is required for transcriptional control, and the relative stabilities of the antiterminator and terminator conformations are thought to be an important parameter (47). Translation initiation is mechanistically based on the sequestration of the ribosomal binding site located at the junction of aptamer domain and expression platform. The roadblock mechanism that many engineered riboswitches can exert may be an outstanding example in this regard, as the aptamer alone performs the function as such. However, the reported successful applications of the tetracycline or the neomycin aptamer clearly reveal that also sophisticated regulatory mechanisms can be realized by engineered riboswitches in addition to roadblock formation. Furthermore, conformational studies on several riboswitches show that ligand induced adaption of the binding pocket region also results in subtle conformational changes in remote aptamer regions and that the adjustment even propagates to the expression platform (48). These subtle changes are often sufficient to mediate riboswitch activity and include both tertiary structure adjustments and changes of base pairings in secondary structure.

Beyond binding kinetics, the observed levels of Mg^2+^ dependence provide further insights. Especially at the lowest analyzed Mg^2+^ concentration, the binding affinity of **A** is substantially affected by the presence of cations. Starting from 0.5 mM Mg^2+^ to 1.5 mM, the *K*_D_ value is reduced by a factor of >50. Consequently, a steep transition from non-binding to binding conformations takes place, just below the physiological range. Fitting the aptamer data to the Hill equation yields a Hill coefficient of 4.6, suggesting that several distinct metal binding sites may be involved in folding into a compact binding-competent structure. The observed effect of Mg^2+^ on the conformation of aptamer **A** is in line with the model that has been proposed for the tetracycline-binding aptamer, which is also incapable of ligand binding in the absence of Mg^2+^ (38). However, the tetracycline-binding aptamer can be applied as a functional riboswitch *in vivo*, in contrast to aptamer **A**. A pronounced Mg^2+^-dependent binding affinity has also been reported for the active theophylline-binding aptamer (49), so this behaviour does not conclusively explain the inactivity of aptamer **A**. In contrast, the *K*_D_ values of the *in vivo* active candidates **preRS** and **RS** are almost independent of Mg^2+^ at concentrations higher than 0.5 mM. This binding behaviour is similar to the neomycin riboswitch, whose binding is also independent of divalent cations. Aptamers in general clearly depend on the availability of divalent ions for binding and the extent of this dependence may vary between the individual aptamers. However, this cannot fully explain the observed differences in the *in vivo* activity of the aptamers explored here.

In sum, the data for the different angles explored here provide a consistent explanation why only a few *in vitro* selected aptamers are suitable for the design of synthetic riboswitches. Only if the two-step binding mechanism with nearly irreversible binding leads to the formation of a ligand-aptamer-complex that has an extended lifetime, is the aptamer thought to function as a gene regulatory element. This finding also explains why only very few such aptamers have been found to date. In the classic SELEX procedure, the ligand is coupled to a solid support. Aptamer candidates that bind to the ligand are usually specifically eluted by adding the free ligand. Candidates that have a longer lifetime of the ligand-bound state thus fail to elute. This is convincingly demonstrated by the deep sequencing data in Figure 1C. Despite a *K*_D_ in the similar range, candidate **A** is 400 times more enriched than the riboswitch precursor **preRS**.

Based on these results, it is now possible to adjust the selection strategy for better enrichment of aptamers with the desired properties. Extended elution times or unspecific elution, or a short pre-elution step with the specific ligand to remove species that show good binding properties but also a fast back-reaction rate, are possibilities to select specifically for the desired properties. However, we see the most promising route in an alternative SELEX strategy, the Capture-SELEX (8). Here the RNA pool is immobilized instead of the ligand. Elution is performed with the free ligand and only aptamers that detach from the immobilization via a capture oligonucleotide and bind the free ligand are recovered. We have recently shown that the chance of finding regulatory aptamers with Capture-SELEX is increased compared to a classical SELEX protocol (8). This may be due to the fact that Capture-SELEX also selects for conformational switching, but the kinetic properties described here certainly contribute to this effect. A slow back reaction of the aptamers bound to the immobilized ligand will prevent efficient elution and consequently enrichment during a classical SELEX procedure; however, it is not an issue for Capture-SELEX.

To conclude, this work enabled us to shed light on an enduring conundrum of synthetic riboswitch design, i.e. why only a very small proportion of the many small molecule-binding aptamers can be used to create synthetic riboswitches. The use of such RNA-based regulators is becoming more and more important, not only as versatile control elements of gene regulation, but also as biosensors to optimize the metabolite formation of synthetic pathways or as low-cost diagnostics for the detection of contaminations. The findings reported here should therefore fundamentally advance the field of RNA-based switches.

## Supplementary Material

gkab166_Supplemental_File
