# Supplementary material for: What defines a synthetic riboswitch? – Conformational dynamics of ciprofloxacin aptamers with similar binding affinities but varying regulatory potentials
Source: Nucleic Acids Res. 2021 Mar 27;49(7):3661–71. doi: 10.1093/nar/gkab166 (PMC8053125; doi:10.1093/nar/gkab166)
Supplement: gkab166_Supplemental_File [file gkab166_supplemental_file.docx]

**Supporting Information**

**What defines a synthetic riboswitch? – Conformational dynamics of non-related ciprofloxacin aptamers with varying regulatory potential**

Christoph Kaiser^1^, Jeannine Schneider^2^, Florian Groher^2^, Beatrix Suess^2,3*^, Josef Wachtveitl^1*^

^1^ Institut für Physikalische und Theoretische Chemie, Goethe-Universität Frankfurt, Max-von-Laue-Straße 8, D-60438 Frankfurt am Main, Germany

^2^ Fachbereich Biologie, Technische Universität Darmstadt, Schnittspahnstraße 10, D-64287 Darmstadt, Germany

^3^ Centre for Synthetic Biology, Technische Universität Darmstadt

**Table of contents**

Time-correlated single photon counting 2

Stopped-flow data processing 3

Kinetic analysis of the stopped-flow data 4

References 12

**Time-correlated single photon counting**

The multi-exponential fitting of the acquired data was carried out with FluoFit Pro 4.6 (PicoQuant) (1). As each of the recorded fluorescence decay curves is convolved with the instrumental response function (IRF), the traces were fitted with the exponential reconvolution function 1.

| $\boldsymbol{I}\left( \boldsymbol{t} \right)\boldsymbol{=}\int_{\boldsymbol{-\infty}}^{\boldsymbol{t}} \boldsymbol{IRF}\left( \boldsymbol{t´} \right)\sum_{\boldsymbol{i=1}}^{\boldsymbol{n}} \boldsymbol{A}_{\boldsymbol{i}}\boldsymbol{e}^{\boldsymbol{-}\frac{\boldsymbol{t-}\boldsymbol{t}_{\boldsymbol{i}}}{\boldsymbol{\tau}_{\boldsymbol{i}}}}\boldsymbol{dt´}$ | (1) |
| --- | --- |

For the fitting procedure, a minimum number *n* of lifetimes τ_i_ was applied. Here, *A_i_* represents the amplitude of the *i*-th component and the fitting was iteratively optimised to obtain a χ^2^ value close to 1.

In addition to the CFX@RNA measurements, the ligand CFX was also measured in buffered solution with the different salt components present. The concentrations were similar to those applied in the SELEX buffer.

**
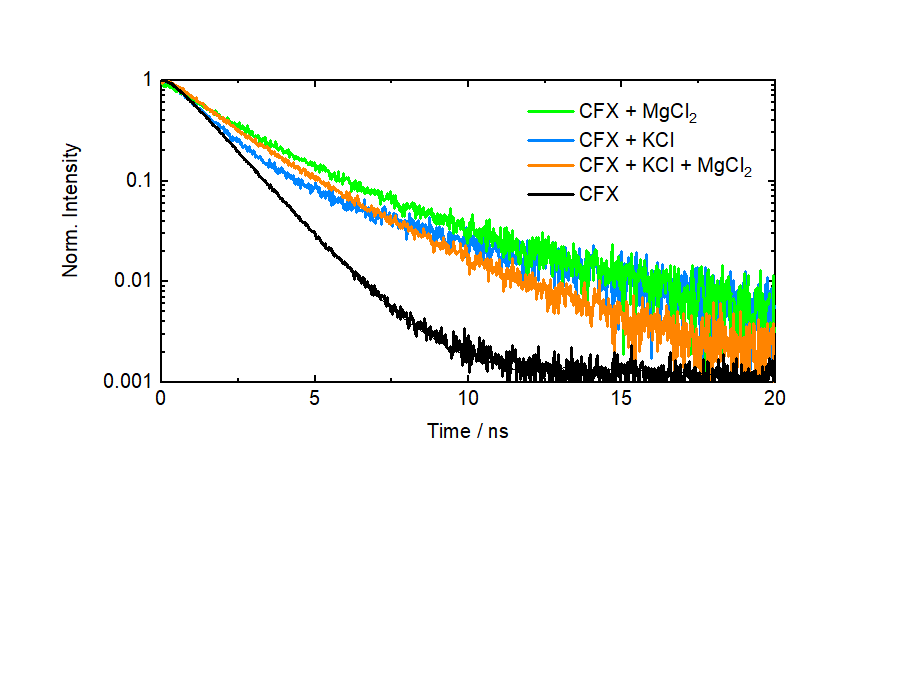
**

**Figure S1.** Normalised fluorescence decay curves of the pure ligand CFX in HEPES buffered solution (black) and in presence of the salts MgCl_2_ (green), KCl (blue) and both salts (orange) at the concentrations applied in the SELEX protocol and the stopped-flow experiments.

**Table S1:** Fluorescence lifetimes τ in ns and corresponding amplitudes A in percent, determined through multi-exponential fitting.

|  | τ_1_ / ns (A / %) | τ_2_ / ns (A / %) | τ_av_ / ns | χ^2^ |
| --- | --- | --- | --- | --- |
| CFX | 1.28 ± 0.01 (100) | **---** | **---** | 1.092 |
| CFX + KCl | 1.22 ± 0.02 (91 ± 2) | 6 ± 0.2 (9 ± 4) | 1.7 | 1.001 |
| CFX + MgCl_2_ | 1.85 ± 0.04 (86 ± 3) | 5.9 ± 0.3 (14 ± 5) | 2.4 | 1.099 |
| CFX + KCl + MgCl_2_ | 1.28 ± 0.02 (65 ± 2) | 2.92 ± 0.04 (35 ± 2) | 1.9 | 1.003 |

**Stopped-flow data processing**

The ligand CFX was supplied in excess relative to the specific RNAs (2, 4, 6, 8 and 10 equivalents = eq), which resulted in pseudo-first order complex formation. The saturation of the RNAs with supplied ligand resulted in a similar signal amplitude for each measurement series. Approximately 30 single transients were recorded for each concentration of CFX (Fig. S2A). All obtained datasets were offset-corrected to account for the residual fluorescence of the free ligand and averaged. The averaged curve was then normalised to compensate for variations in the RNA concentration. The traces were recorded from 20 ms before the mixing of the particular components and the hard stop timing was set to 40 ms. Considering the dead time of the stopped-flow device, the time axis was corrected for the starting point of the kinetics. The data were acquired with 5600 timeframes of 50 μs, then 2000 frames of 200 μs and 400 frames of 1 ms. By averaging the traces, the curves shown below were obtained with equally spaced datapoints.


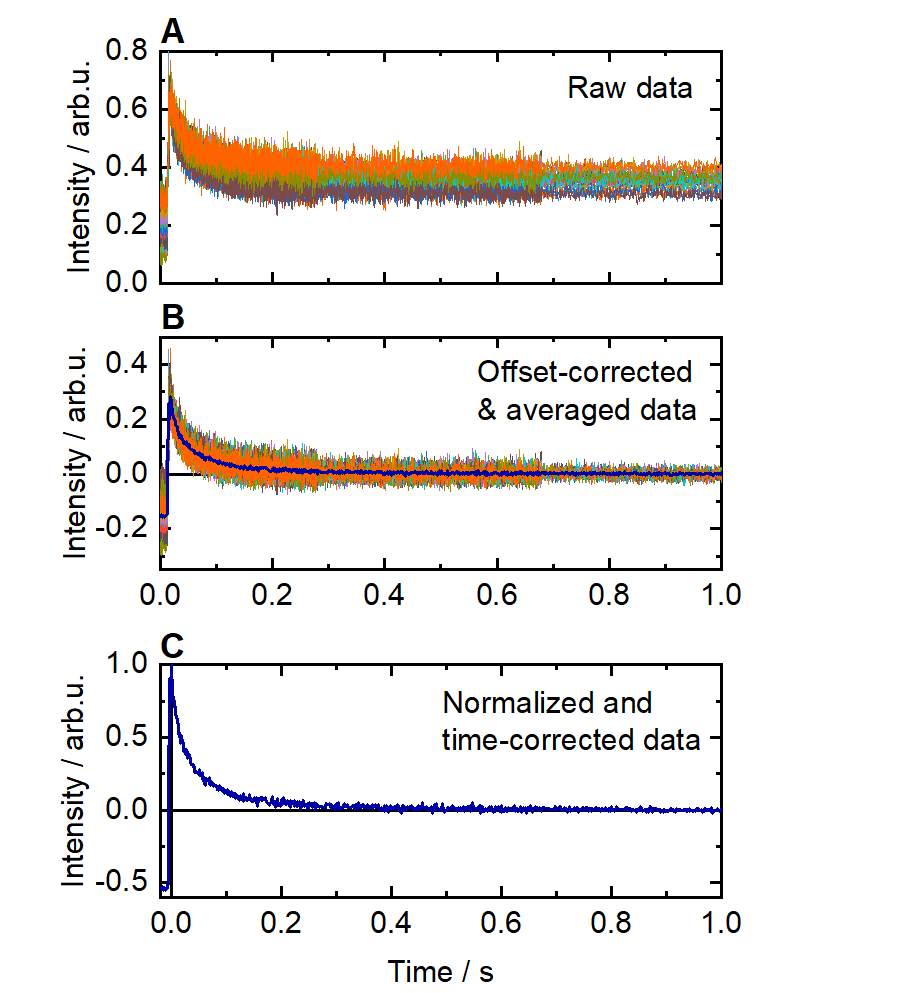


**Figure S2.** Data processing routine, exemplified for the stopped-flow experiment of the aptamer A mixed with 2 eq CFX. (**A**) Raw data acquired with predefined time frames. The non-zero fluorescence detected after equilibration accounts for the residual emission from unbound CFX. (**B**) The raw data have been offset-corrected by subtracting the mean value of the last 50 datapoint for each individual trace. The traces were then averaged (solid blue curve) and the datapoint spacing was adjusted to be equidistant. (**C**) The averaged curve was finally normalised at the starting point of the binding kinetics and the time axis was shifted to the respective point.

**Kinetic analysis of the stopped-flow data**

The kinetic fitting of the individual datasets was performed with the software DynaFit4 (2). The observed time-dependent signal S(t) was approximated with the general fit function given in equation 2.

| $\boldsymbol{S}\left( \boldsymbol{t} \right)\boldsymbol{=}\boldsymbol{S}_{\boldsymbol{0}}\boldsymbol{+}\sum_{\boldsymbol{i=1}}^{\boldsymbol{n}} \boldsymbol{r}_{\boldsymbol{i}}\boldsymbol{c}_{\boldsymbol{i}}\boldsymbol{(t)}$ | (2) |
| --- | --- |

Here, *S_0_* represents the starting point of the signal and *n* corresponds to the number of molecular species engaged in the particular model that contribute to the detected signal. Hence, *n* = 3 for the two-step models 3-5, as the pure ligand CFX is involved as well as the intermediate species CFX@RNA* and the final complex CFX@RNA. The parameter *r_i_* (response coefficient) gives the relative contributions of the involved *i*th species and the respective binding steps to the decrease of the ligand fluorescence. The values were determined to be approximately 50% for each of the steps in the induced-fit models. Last, the concentrations *c_i_(t)* of the individual species were calculated from their initial concentrations by solving a system of differential equations based on the kinetic models. Only the rate constants were treated as free parameters and other parameters like e.g. the response coefficients were optimised at first step and then fixed.

The differential equations corresponding to the particular models are shown below as well the determined kinetic parameters obtained from the fitting routine (Tables S2–5). Analytical solutions of the respective differential equations are reported elsewhere (3). The resulting fit qualities of the statistical models were then subjected to a comparative evaluation by calculation of the Akaike Information criterion (AIC)(4) and the Bayesian information criterion (BIC)(5) following the equations 3 and 4, respectively.

| $\boldsymbol{AIC=}\left[ \boldsymbol{-log}\left( \boldsymbol{L}_{\boldsymbol{p}} \right)\boldsymbol{+2 p} \right]$ | (3) |
| --- | --- |
| $\boldsymbol{BIC=}\left[ \boldsymbol{-log}\left( \boldsymbol{L}_{\boldsymbol{p}} \right)\boldsymbol{+}\log\left( \boldsymbol{n} \right)\boldsymbol{p} \right]$ | (4) |

*L_p_* represents the likelihood function maximum as a measure of the particular fit quality, *p* is the number of free parameters and *n* is the sample size. Both criteria provide a trade-off between the fit accuracy and the simplicity of an applied model, as the number of parameters is penalized by a weighting factor. Consequently, the penalty for an increased number of free parameters is more severe in case of the BIC. The values of the information criteria should be very small or even zero for the model with the highest probability. Typically, the difference values to the most probable model ΔAIC and ΔBIC are calculated. The obtained curve fits are collected in the Figure S3-5 and the model selection criteria are shown in Table S6.

| Model 1 | 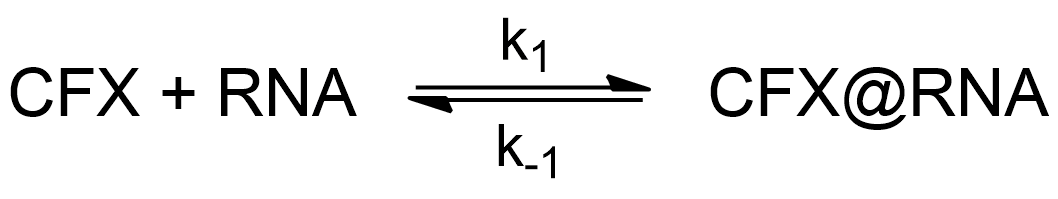 |
| --- | --- |

$$\frac{d[CFX]}{dt}=\frac{d[RNA]}{dt}=-\frac{d[CFX@RNA]}{dt}=-k_{1}\left[ CFX \right][RNA]+k_{-1}\left[ CFX@RNA \right]$$

**Table S2:** Rate constants of the reversible one-step model 1, determined by kinetic analysis for the three candidates **A**, **preRS** and **RS**.

|  | k_1_ / μM^-1^s^-1^ | k_-1_ / s^-1^ | RMSD |
| --- | --- | --- | --- |
| A | 7.78 ± 0.09 | 0.33 ± 0.03 | 0.044783 |
| preRS | 3.61 ± 0.03 | 0.09 ± 0.01 | 0.030391 |
| RS | 5.21 ± 0.04 | <10^-6^ | 0.03298 |

| Model 2 | 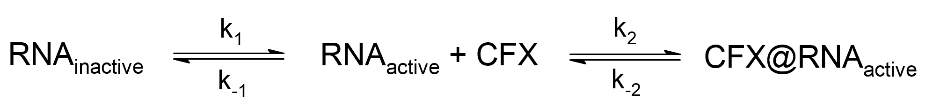 |
| --- | --- |

$$\frac{d\left[ CFX \right]}{dt}=\frac{d\left[ {RNA}_{active} \right]}{dt}$$

$$=k_{1}\left[ {RNA}_{inactive} \right]-\left( k_{-1}+k_{2} \right)\left[ CFX \right]\left[ {RNA}_{active} \right]+k_{-2}[CFX@{RNA}_{active}]$$

$$\frac{d[CFX@RNA]}{dt}=k_{2}\left[ CFX \right]\left[ RNA \right]-k_{-2}[CFX@RNA]$$

**Table S3:** Rate constants of the conformational selection model 2, determined by kinetic analysis for the three candidates **A**, **preRS** and **RS**.

|  | k_1_ / s^-1^ | k_-1_ / μM^-1^s^-1^ | k_2_ / μM^-1^s^-1^ | k_-2_ / s^-1^ | RMSD |
| --- | --- | --- | --- | --- | --- |
| A | 14.6 ± 1.6 | 8 ± 1 | 9.6 ± 0.2 | < 10^-7^ | 0.043078 |
| preRS | 10.1 ± 0.8 | 4.7 ± 0.5 | 4.48 ± 0.07 | 0.034 ± 0.007 | 0.028402 |
| RS | 68 ± 10 | 24 ± 6 | 6.8 ± 0.3 | < 10^-5^ | 0.032326 |

| Model 3 | 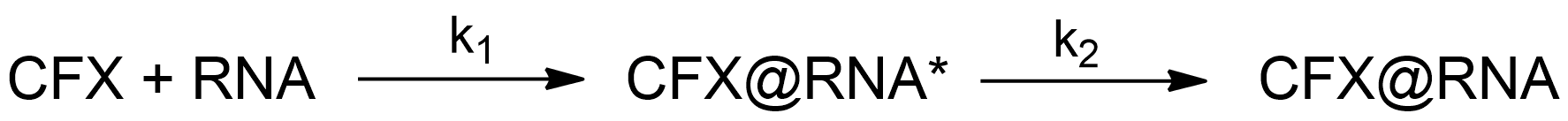 |
| --- | --- |

$$\frac{d[CFX]}{dt}=\frac{d[RNA]}{dt}=-k_{1}\left[ CFX \right]\left[ RNA \right]$$

$$\frac{d[{CFX@RNA}^{*}]}{dt}=k_{1}\left[ CFX \right]\left[ RNA \right]-k_{2}[{CFX@RNA}^{*}]$$

$$\frac{d[CFX@RNA]}{dt}=k_{2}[{CFX@RNA}^{*}]$$

**Table S4:** Rate constants of model 3 with two irreversible steps, determined by kinetic analysis for the three candidates **A**, **preRS** and **RS**.

|  | k_1_ / μM^-1^s^-1^ | k_2_ / s^-1^ | RMSD |
| --- | --- | --- | --- |
| A | 9.7 ± 0.2 | 4.9 ± 0.2 | 0.041642 |
| preRS | 3.79 ± 0.03 | 1.9 ± 0.2 | 0.030244 |
| RS | 6.87 ± 0.06 | 16.4 ± 0.5 | 0.030797 |

| Model 4 | 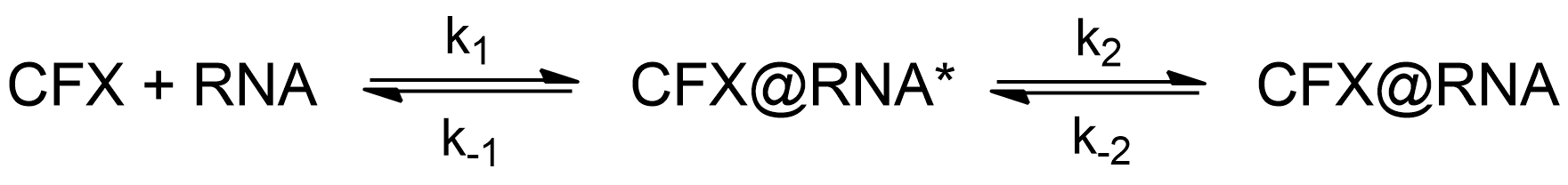 |
| --- | --- |

$$\frac{d[CFX]}{dt}=\frac{d[RNA]}{dt}=-k_{1}\left[ CFX \right][RNA]+k_{-1}\left[ {CFX@RNA}^{*} \right]$$

$$\frac{d[{CFX@RNA}^{*}]}{dt}=k_{1}\left[ CFX \right]\left[ RNA \right]-k_{-1}\left[ {CFX@RNA}^{*} \right]-k_{2}\left[ {CFX@RNA}^{*} \right]+k_{-2}[CFX@RNA]$$

$$\frac{d[CFX@RNA]}{dt}=k_{2}\left[ {CFX@RNA}^{*} \right]-k_{-2}[CFX@RNA]$$

**Table S5:** Rate constants of model 4 with two reversible steps, determined by kinetic analysis for the three candidates **A**, **preRS** and **RS**.

|  | k_1_ / μM^-1^s^-1^ | | k_-1_ / s^-1^ | k_2_ / s^-1^ | k_-2_ / s^-1^ | RMSD | |
| --- | --- | --- | --- | --- | --- | --- | --- |
| A | 10 ± 0.2 | | 1.6 ± 0.2 | 4 ± 0.2 | 2.3 ± 0.2 | 0.041255 | |
| preRS | 4.33 ± 0.06 | | 5.7 ± 0.5 | 13.2 ± 0.8 | 0.06 ± 0.03 | 0.028187 | |
| RS | 7.6 ± 0.1 | | 3.1 ± 0.6 | 23 ± 1 | 0.27 ± 0.05 | 0.030741 | |
|  | |  | | | | |  |

| Model 5 | 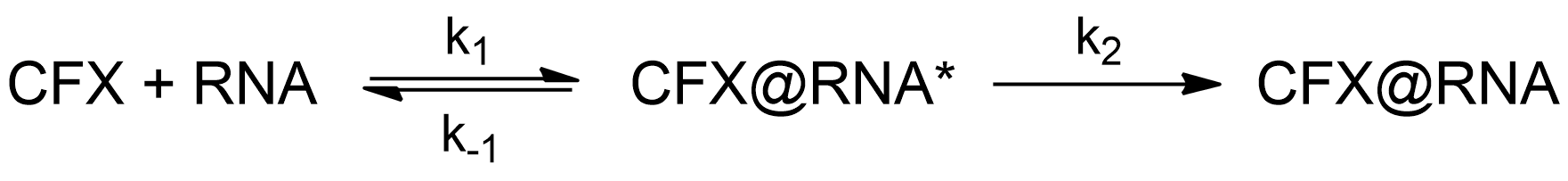 |
| --- | --- |

$$\frac{d[CFX]}{dt}=\frac{d[RNA]}{dt}=-k_{1}\left[ CFX \right][RNA]+k_{-1}\left[ {CFX@RNA}^{*} \right]$$

$$\frac{d[{CFX@RNA}^{*}]}{dt}=k_{1}\left[ CFX \right]\left[ RNA \right]-k_{-1}\left[ {CFX@RNA}^{*} \right]-k_{2}\left[ {CFX@RNA}^{*} \right]$$

$$\frac{d[CFX@RNA]}{dt}=k_{2}\left[ {CFX@RNA}^{*} \right]$$

**Table S6:** Rate constants of model 5 with a reversible first step followed by an irreversible second step, determined by kinetic analysis for the three candidates **A**, **preRS** and **RS**.

|  | k_1_ / μM^-1^s^-1^ | k_-1_ / s^-1^ | k_2_ / s^-1^ | RMSD |
| --- | --- | --- | --- | --- |
| A | 10 ± 0.2 | 0.8 ± 0.2 | 6 ± 0.3 | 0.041582 |
| preRS | 4.31 ± 0.06 | 5.4 ± 0.4 | 12.1 ± 0.7 | 0.028198 |
| RS | 7.62 ± 0.11 | 2.9 ± 0.7 | 23 ± 1 | 0.030736 |


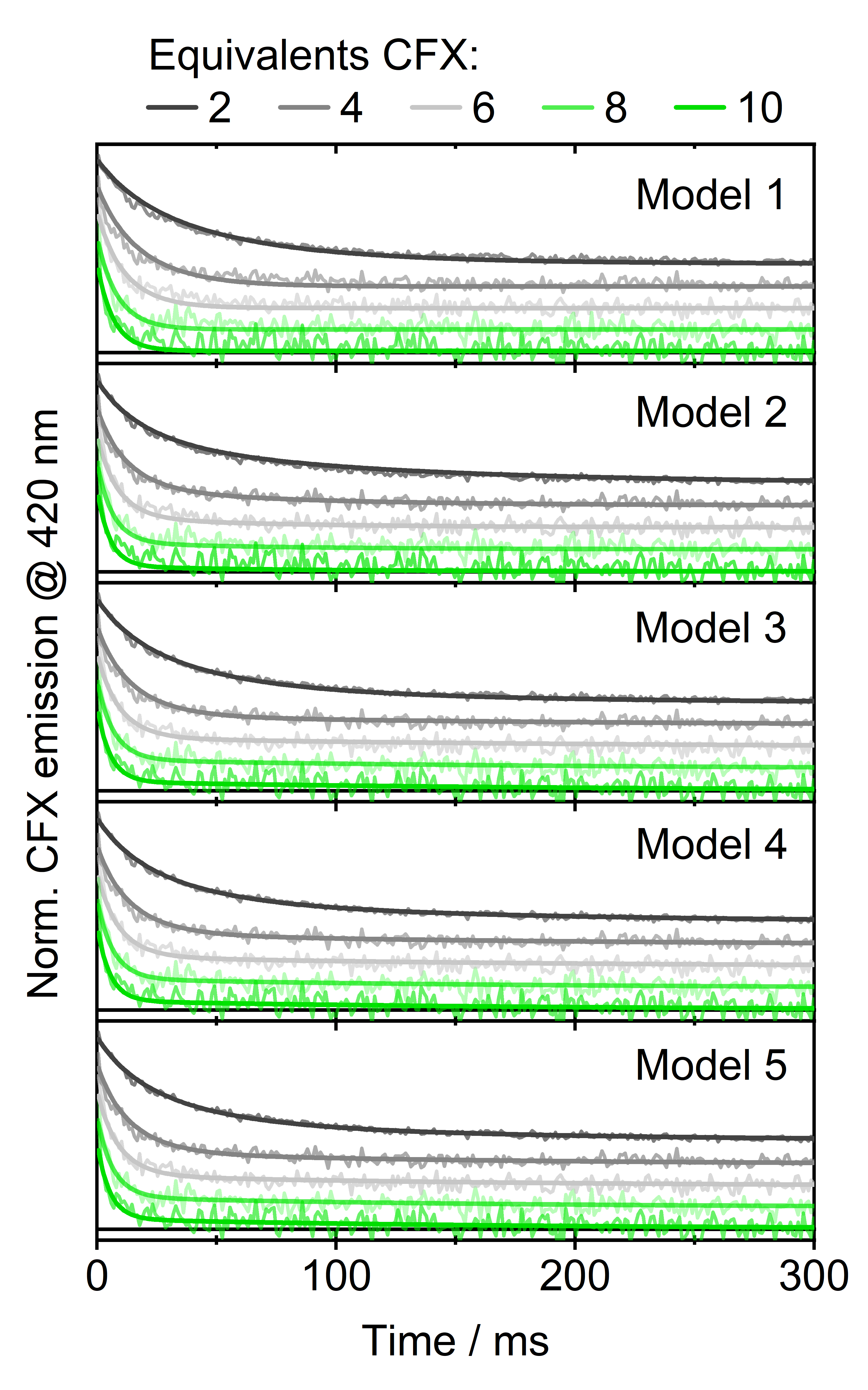


**Figure S3:** Comparison of the fit qualities of the applied kinetic models 1-5 for the aptamer **A**.


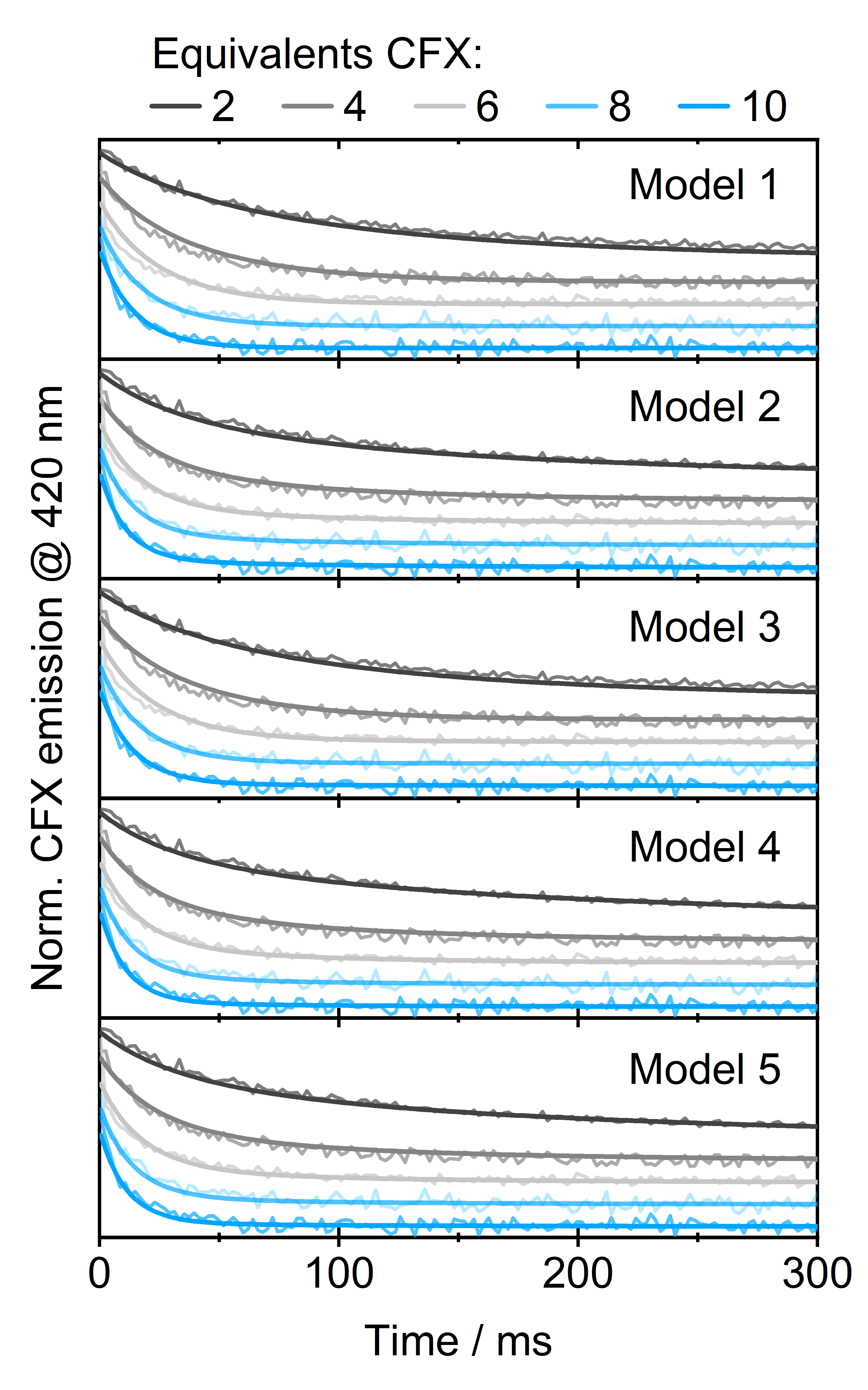


**Figure S4:** Comparison of the fit qualities of the applied kinetic models 1-5 for the potential riboswitch **preRS**.


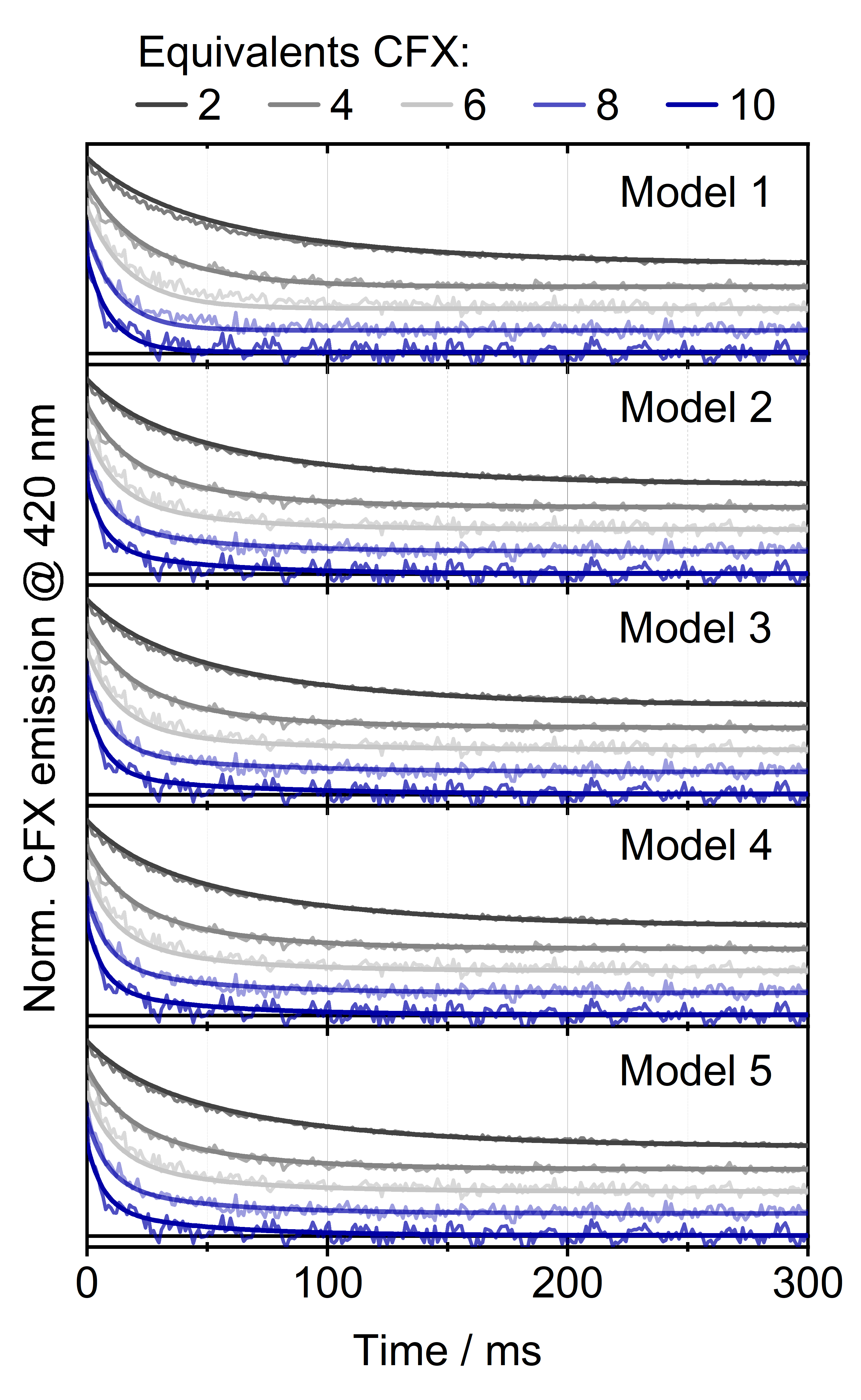


**Figure S5:** Comparison of the fit qualities of the applied kinetic models 1-5 for the potential riboswitch **RS**.

**Table S7:** Summary of the comparative analyses of the ligand-binding kinetics of **A**, **preRS** and **RS**. The corresponding number of free parameters p is given for each of the models 1-5 and the obtained RMSD of the fit. The relative probabilities of the models are reflected by the lowest values of the likelihood criteria ΔAIC and ΔBIC.

|  | **Model** | **p** | **RMSD** | **ΔAIC** | **ΔBIC** |
| --- | --- | --- | --- | --- | --- |
| **A** | **1** | 2 | 0.044783 | 801 | 788 |
|  | **2** | 4 | 0.043078 | 424 | 424 |
|  | **3** | 2 | 0.041642 | 88 | 75 |
|  | **4** | 4 | 0. 041255 | 0 | 0 |
|  | **5** | 3 | 0.041582 | 75 | 69 |
| **preRS** | **1** | 2 | 0.030391 | 742 | 733 |
|  | **2** | 4 | 0.028402 | 75 | 80 |
|  | **3** | 2 | 0.030244 | 694 | 685 |
|  | **4** | 4 | 0.028187 | 0 | 5 |
|  | **5** | 3 | 0.028198 | 2 | 0 |
| **RS** | **1** | 2 | 0.03298 | 701 | 694 |
|  | **2** | 4 | 0.032326 | 505 | 511 |
|  | **3** | 2 | 0.030797 | 18 | 11 |
|  | **4** | 4 | 0.030741 | 4 | 10 |
|  | **5** | 3 | 0.030736 | 0 | 0 |

**References**

1. Enderlein,J. and Erdmann,R. (1997) Fast fitting of multi-exponential decay curves. *Opt. Commun.*, **134**, 371–378.

2. Kuzmic,P. (1996) Program DYNAFIT for the analysis of enzyme kinetic data: application to HIV proteinase. *Anal. Biochem.*, **237**, 260–273.

3. Andraos,J. (1999) A streamlined approach to solving simple and complex kinetic systems analytically. *J. Chem. Educ.*, **76**, 1578.

4. Akaike,H. (1974) A New Look at the Statistical Model Identification. *IEEE Trans. Automat. Contr.*, **19**, 716–723.

5. Myung,J.I., Tang,Y. and Pitt,M.A. (2009) Evaluation and Comparison of Computational Models. *Methods Enzymol.*, **454**, 287–304.
